# Education Research: Impact of Burnout on Neurology Residents and Research Fellows in Europe

**DOI:** 10.1212/NE9.0000000000200035

**Published:** 2022-11-29

**Authors:** Giovanni Di Liberto, Giuseppina Baldizzi, Vanessa Carvalho, Luca Cuffaro, Anna Sauerbier, Lisa Klingelhoefer, Nina Vashchenko, Lucia Pavlakova, Anja Sander, Marianne de Visser, Paul A.J.M. Boon, Elena Moro, Claudio Bassetti

**Affiliations:** From the Department of Clinical Neurosciences (G.D.L., G.B.), Centre Hospitalier Universitaire Vaudois and University of Lausanne, Switzerland; Department of Neurosciences and Mental Health (Neurology) (V.C.), Hospital Santa Maria-CHLN, and Centro de Estudos Egas Moniz (V.C.), Faculdade de Medicina, Universidade de Lisboa, Portugal; Department of Health Sciences (L.C.), Clinical Neurology Unit, ASST Santi Paolo & Carlo, Milan, Italy; Department of Neurology (A. Sauerbier), Faculty of Medicine and University Hospital Cologne, University of Cologne; Department of Neurology (L.K.), Technical University Dresden, Germany; University Hospital of Neurological Disorders (N.V.), Sechenov First Moscow State Medical University, Russia; European Academy of Neurology (L.P., A. Sander), Head Office, Vienna, Austria; Department of Neurology (M.d.V.), Academic Medical Center, Amsterdam, the Netherlands; Department of Neurology (P.A.J.M.B.), Ghent University Hospital, Belgium; Division of Neurology (E.M.), CHU of Grenoble, Grenoble Institute of Neurosciences, Grenoble Alpes University, France; and Department of Neurology (C.B.), Inselspital, University of Bern, Switzerland.

## Abstract

**Background and Objectives:**

To investigate the prevalence of burnout profiles and their contributing factors in European neurology residents, junior neurologists, and research fellows.

**Methods:**

The members of the Resident and Research Fellow Section (RRFS) of the European Academy of Neurology were surveyed using standardized instruments for burnout, job satisfaction, social support, depression, work-related fatigue, work-life integration, and impact of life events, from September 1, 2020, to January 6, 2021.

**Results:**

The response rate was 23.1% (332 responders of 1,439 contacted RRFS members); the median age of participants 30 years, with 61.5% being female. Our analysis revealed that 73.9% of the responders showed a perturbation in the Maslach Burnout Inventory dimensions, thus fulfilling the criteria for a dysfunctional phenotype within the burnout spectrum: burnout (22.6%), overextended (26.1%), ineffective (21.0%), and disengaged (4.1%). Multivariate logistic regression analysis indicated that elevated number of night shifts per month, work-related fatigue, and low professional satisfaction are independent risk factors for burnout. Being a certified neurologist, working in an academic environment, and a higher job satisfaction are associated with the engaged profile, a productive state characterized by high energy, strong involvement, and a sense of efficacy.

**Discussion:**

Burnout experience is highly prevalent among neurology residents, junior neurologists, and research fellows in Europe. The profile-based approach in this survey captures the multifaceted nature of burnout experience, therefore highlighting mitigating strategies that can be tailored to the distinct dysfunctional burnout profile.

Neurology residents and research fellows are fundamental for the functioning of health care and academia.^1^ Their physical and mental well-being is often shaped by several individual and situational factors which can increase the burnout risk.^2,3^

Burnout is a multifaceted psychological syndrome emerging as a dysfunctional adaptation to chronic job-related and unsuccessfully managed interpersonal stressors. According to the *International Classification of Diseases, 11th revision*, this occupational phenomenon is characterized by an overwhelming fatigue and emotional exhaustion, feelings of cynicism, detachment from the job, a sense of ineffectiveness, and lack of personal accomplishment.^4,5^

Physicians with burnout may experience a wide range of work-life issues including medical errors,^6^ conflicts, absenteeism, poor social life interactions, as well as an increased risk of depression and suicide.^7,8^

Given the variable severity, individuals can manifest a perturbation in multiple Maslach Burnout Inventory (MBI) dimensions in the full-blown burnout or rather in a single dimension,^9^ thus giving rise to distinct profiles identified as overextended (high emotional exhaustion), disengaged (high cynicism), and ineffective (low efficacy). By contrast, the engaged profile represents the positive antithesis to burnout and can be considered as a productive state characterized by high energy, strong involvement, and a sense of efficacy.^10^

Recent studies in this field have shown a substantial variability in the burnout prevalence among physicians.^11^ This issue emerged from variations in burnout definitions, assessment methods, and study quality, therefore precluding definitive conclusions about its prevalence and highlighting the importance of standardized measurement tools such as a profile-based approach based on the MBI.^11^

Burnout prevalence appears to be particularly high among neurology residents as compared with specialists and other physicians,^2,12-14^ although the underlying reasons remain largely unknown.

Elucidating the factors contributing to this phenomenon is essential to preserve the current neurologic workforce overtime, given the growing neurologic care demand in the next decades^15^ and the lack of data on this topic.

In this study, we conducted an online survey to assess the burnout prevalence and its contributing factors among European neurology residents, junior neurologists, and research fellows with the aim to shed light on their workplace well-being and identify factors contributing to their work-related distress.

## Methods

### Study Population

The population of interest included all the neurology residents, junior neurologists (up to 3 years from their certification), and research fellows working in Europe and who were members of the European Academy of Neurology (EAN)-Resident and Research Fellow Section (RRFS) at the time of the survey by September 1, 2020 (n = 1,439). Assuming a population size of 1,439 members and aiming for a 95% CI and a 5% margin of error, we required a minimum sample size of 303 participants. Individuals in the sample were anonymized and e-mailed with a link to the online version of the survey from September 1, 2020, to January 6, 2021. Nonresponders received 2 additional e-mail reminders. Participants who completed the survey were eligible for a drawing of 10 gift vouchers of 50 €.

### Standard Protocol Approvals, Registrations, and Participant Consent

Consent to participate in the study was implied by completing the survey. After response collection through an online platform, anonymized data were used for interpretation and analysis. This study was reviewed and granted exempt status by the Human Research Ethics Committee of the Canton Vaud (Switzerland). The RRFS e-mail was available in case the participants asked for psychological support during or after completion of the questionnaire. To retain confidentiality, a dedicated person within the EAN head office was responsible for treating these requests and address the participants to their local psychiatric emergency ward offering prompt on-site consultations.

### Study Measures

#### Questionnaire

The survey was based on existing literature and consisted of 160 items covering demographics, professional characteristics, and standardized instruments to measure burnout, job satisfaction, social support, depression, work-related fatigue, functioning in professional and private environments, and stressful life events (eAppendix 1). Questions 10 and 13 were extracted from the American Academy of Neurology survey^16^ and reproduced with permission (eAppendix 2).

#### Maslach Burnout Inventory

Burnout was measured using the 22-item MBI–Human Services Survey (MBI-HSS).^17^ The MBI-HSS addresses the 3 dimensions of burnout: emotional exhaustion, cynicism (or depersonalization), and efficacy (or personal accomplishment). Profiles were defined by taking advantage of standardized (*z*) values for the occupational subgroup medicine (n = 1,104),^9^ setting the critical boundary for exhaustion at *z* = 27.0 (mean + [SD × 0.5]), for cynicism at *z* = 14.0 (mean + [SD × 1.25]), and efficacy at *z* = 37.0 (mean + [SD × 0.1]). Therefore, we considered as engaged, individuals with a score of exhaustion <27, cynicism <14, and efficacy ≥37; ineffective, individuals with a score of efficacy <37, with exhaustion <27 and cynicism <14; overextended, individuals with a score of exhaustion ≥27 with cynicism <14; disengaged, individuals with a score of cynicism ≥14 and exhaustion <27; burnout, individuals with a score of exhaustion ≥27 and cynicism ≥14.

#### Job Satisfaction Survey

Job satisfaction was measured with the Job Satisfaction Survey (JSS), a 36-item scale exploring 9 facets of job satisfaction^18^: pay, promotion, supervision, fringe benefits, contingent rewards (performance-based rewards), operating procedures (required rules and procedures), coworkers, nature of work, and communication. The total scores range from 36 to 216, indicating dissatisfaction (36–108), satisfaction (144–216), and ambivalence (109–143). For the 4-item subscales, a mean item response indicates satisfaction (≥4), dissatisfaction (≤3), and ambivalence (3–4).

#### Multidimensional Scale of Perceived Social Support

The Multidimensional Scale of Perceived Social Support (MSPSS)^19^ is a 12-item scale designed to measure perceptions of social support from 3 sources: family, friends, and a significant other. According to their mean scores, we can identify a low support (<3), moderate support (3–5), or high support (>5).

#### Patient Health Questionnaire-9

The Patient Health Questionnaire-9 (PHQ-9)^20^ assessed the depression risk. According to the score, depression can be considered: absent (<4), mild (5–9), moderate (10–14), moderately severe (15–19), or severe (≥20).

#### Three-Dimensional Work Fatigue Inventory

Work-related fatigue is a multidimensional psychological construct characterized by the depletion of physical, mental, and emotional resources and resulting in extreme tiredness and reduced functional capacity.^21^ The Three‐Dimensional Work Fatigue Inventory (3D‐WFI) is an 18-item scale evaluating physical, mental, and emotional work fatigue based on the depletion of these specific resources.^21^ According to the score, fatigue can be considered: severe (≥40), moderate (20–40), or low (<20).

#### Work and Social Adjustment Scale

The Work and Social Adjustment Scale (WSAS) is a 5-item tool assessing the impact of a person's mental health condition on their ability to function in terms of work, home management, social leisure, private leisure, and personal or family relationships.^22^ According to the score, this scale indicates severe impairment (>20), moderate impairment (10–20), or low impairment (<10).

#### Life Change Index

The Life Change Index Scale (LCI)^23^ measures stressful life events happened in the last year that could contribute to illness. According to the score, this scale indicates a severe risk of illness (>300), a moderate risk (150–299), or a low risk (<150).

### Statistical Analysis

Standard descriptive statistics were used to characterize responders. To compare 2 groups, an unpaired 2-tailed Student *t* test (parametric) or a Mann-Whitney test (nonparametric) was used for continuous variable and the χ^2^ test for categorical variables. To compare multiple groups, the one-way analysis of variance test (with equal variance) or Kruskal-Wallis test (for unequal variance) were used. Linear regression was used to evaluate the association between 2 variables. Multivariate analysis with binary logistic regression was performed to identify factors predicting either burnout or engaged profile. Our multivariate analyses included the following variables: JSS score, MSPSS score, night shifts per month, 3D-WFI score, PHQ-9 score, and certified neurologist status.

Multivariate analysis with binary logistic regression was also performed to identify factors predicting the low-income group including the following variables in the model: low-income group, sex, geographic region, academic workplace, being a certified neurologist, and working hours per week.

Statistical analysis was performed in GraphPad Prism9 and R.

### Data Availability

Anonymized data not published within this article will be made available by request from any qualified investigator.

## Results

### Representativeness and Demographics

The survey was sent to 1,439 RRFS members. We collected 332 responses from September 1, 2020, to January 6, 2021. Complete information about sex, age, and geographic region was available for 903 responders, while information about career stage was available for 822 of them. Responders did not differ significantly from nonresponders regarding age, sex, geographic region (based on United Nations geoscheme), and career stage (Table 1).

**Table 1:** Representativeness and Demographics

	Responders	Nonresponders	*p* Value
Age, y	n = 314	n = 589	0.061
Mean	30.9	31.3	
Median	30.0	31.0	
SD	3.5	3.3	
Sex, %	n = 314	n = 589	0.880
Female	61.5	61.0	
Region, %	n = 314	n = 589	0.244
Northern Europe	4.8	6.3	
Southern Europe	40.5	40.8	
Eastern Europe	23.3	21.9	
Western Europe	23.3	26.2	
Other	8.3	4.9	
Career stage, %	n = 314	n = 508	0.395
Neurology resident	48.7	39.2	
Neurologist	31.9	31.9	
Research fellow/PhD student	19.8	17.8	

Comparisons tested using Mann-Whitney for continuous variables (age) and χ^2^ for categorical variables (sex, region, career stage). Geographic region was assigned according to the United Nations Geoscheme.

**Figure 1:**
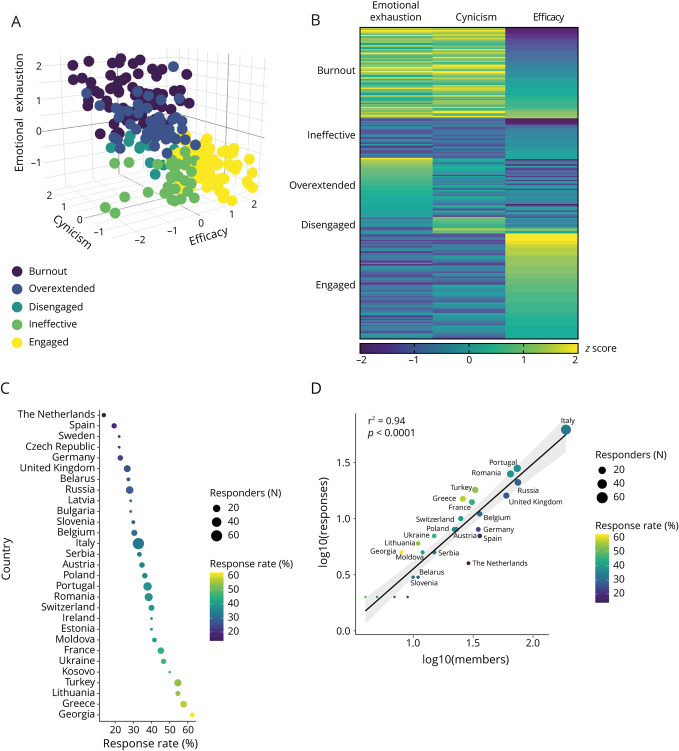
Burnout Profiles and Regional Distribution in Europe (A) Three-dimensional scatter plot illustrating *z*-score distributions for each MBI subscale (emotional exhaustion, cynicism, efficacy), each dot represents a survey responder while burnout profiles are indicated with different color hues (burnout, overextended, disengaged, ineffective, engaged). (B) Heat map illustrating z-scores for each MBI subscale for each burnout profile. (C) Country response rate is depicted by absolute number of response (dot size) and proportion of responses (color scale). (D) Correlation plot of RRFS members and responders per country; each dot represents a country, dot size indicates the absolute number of responses while dot color illustrates the response rate, and axes are expressed in log10 scale. MBI = Maslach Burnout Inventory; RRFS = Resident and Research Fellow Section.

Despite the variability of response rate among different countries (Figure 1C), the number of responders per country correlated with the number of RRFS members per country (*R*^2^ = 0.94, *p* < 0.0001, Figure 1D), thus reflecting the internal representation of members within the RRFS.

Taken together, the sample was generally representative of RRFS composition regarding demographic characteristics.

Among the responders, nearly 50% were neurology residents, while roughly 30% were junior neurologists and 20% research fellows (Table 1). The median age of all participants was 30 years, and 61.5% were female. Women outnumbered men in all the career stages except for neurologists doing a PhD (Table 2).

**Table 2:** Professional Characteristics

	All (n = 314)	Neurology resident (n = 122)	Neurology resident doing a PhD (n = 30)	Neurologist (n = 55)	Neurologist doing a PhD (n = 45)	PhD in neurology research (n = 35)	Post-doc in neurology research (n = 27)
Female/male ratio							
Ratio	1.6	1.7	1.7	2.1	0.9	1.7	1.7
Hours per week							
Mean	49.5	50.6	51.4	47.2	48.6	47.3	50.9
Median	50.0	50.0	50.0	40.0	50.0	45.0	50.0
SD	13.5	13.3	11.6	14.2	11.7	15.5	15.0
Time devoted to clinical practice, %							
Mean	60.6	71.6	54.6	72.7	51.2	34.2	47.4
Median	65.0	75.5	55.0	80.0	50.0	27.5	50.0
SD	26.9	21.2	24.4	21.4	24.5	27.9	23.1
Time devoted to research, %							
Mean	21.2	9.7	21.5	16.6	31.0	58.8	27.8
Median	10.0	5.0	10.0	10.0	25.0	55.0	20.0
SD	24.0	10.7	23.0	16.4	22.9	29.6	20.6
Time devoted to teaching, %							
Mean	5.9	4.5	7.9	6.7	7.8	7.6	11.1
Median	0.0	0.0	5.0	5.0	5.0	5.0	10.0
SD	11.1	10.4	10.9	9.7	12.0	9.7	17.8
Time devoted to administrative work, %							
Mean	10.6	13.2	13.8	13.4	6.9	8.4	11.3
Median	5.0	5.0	10.0	10.0	5.0	10.0	10.0
SD	13.7	16.8	14.1	11.8	8.8	8.1	11.5
Time devoted to other activities work, %							
Mean	1.5	1.4	1.5	2.7	2.9	3.4	2.1
Median	0.0	0.0	0.0	0.0	0.0	0.0	0.0
SD	4.3	3.6	3.5	4.1	7.3	6.2	4.7
Nights on call per month							
Mean	2.9	3.3	3.3	2.8	2.2	1.1	2.0
Median	3.0	3.0	3.0	3.0	2.0	0.0	2.0
SD	3.3	2.3	2.7	3.1	1.9	2.1	2.0
Weekend on call per month							
Mean	1.6	1.8	1.5	1.4	1.4	1.0	1.3
Median	2.0	2.0	2.0	1.0	1.0	0.0	1.0
SD	1.2	1.1	1.0	1.3	1.3	1.3	0.9
Working in university hospital, %							
Proportion	80.3	79.5	96.7	61.8	88.9	88.6	77.8
Affected by the COVID-19 pandemic with increase in workload, %							
Proportion	43.6	37.7	50.0	52.7	51.1	28.6	48.2
Duration of neurology residency or PhD program, y							
Mean	NA	4.7	4.9	NA	3.7	3.4	NA
Median	NA	5.0	5.0	NA	3.0	3.0	NA
SD	NA	0.7	1.1	NA	1.0	0.6	NA
NRP year, %							
NRP1							
Proportion	NA	11.5	10.0	NA	NA	NA	NA
NRP2							
Proportion	NA	13.9	20.0	NA	NA	NA	NA
NRP3							
Proportion	NA	29.5	16.7	NA	NA	NA	NA
NRP4							
Proportion	NA	34.4	23.3	NA	NA	NA	NA
NRP5 or more							
Proportion	NA	10.7	26.7	NA	NA	NA	NA
Time after neurology certification, y							
Mean	NA	NA	NA	1.8	2.2	NA	NA
Median	NA	NA	NA	1.0	2.0	NA	NA
SD	NA	NA	NA	1.4	1.8	NA	NA
Workplace/residency abroad, %							
Proportion	11.5	9.0	10.0	14.3	8.9	25.7	11.1
Annual income, % of responders							
<15,000 €	36.6	36.1	33.3	41.8	37.8	28.6	40.7
15,000–30,000 €	39.2	47.5	36.7	25.5	40.0	40.0	29.6
30,000–45,000 €	13.7	6.6	13.3	23.6	17.8	25.7	3.7
45,000–60,000 €	5.1	4.1	10.0	3.6	4.4	5.7	7.4
60,000–75,000 €	1.6	3.3	0.0	0.0	0.0	0.0	3.7
75,000–90,000 €	2.2	0.8	6.7	0.0	0.0	0.0	14.8
>90,000 €	1.6	1.6	0.0	5.5	0.0	0.0	0.0

Abbreviations: COVID-19 = coronavirus disease 2019; NA = not applicable; NRP = neurology residency programme.

### Professional Demographics

Responders worked a mean of 49.5 hours per week, with a median of 65% of time devoted to clinical practice and 10% of time spent in research (Table 2). Responders occupying full-time clinical positions spent more time on clinical care with a median of 80% of time for neurologists and 75.5% of time for neurology residents, respectively. Neurology residents and neurologists spent a similar number of nights shifts per month and weekends per month (Table 2). Around 80% of the responders worked in a university hospital or laboratory.

Nearly 44% of the participants reported an increase in workload because of coronavirus disease 2019 (COVID-19) pandemic. Slightly more than 10% of the responders were currently working at a different place than their home country, with most of the participants moving to Western European countries to do their training or work (Table 2, eFigure 1A http://links.lww.com/NE9/A9).

Three-quarters of the responders reported an annual income lower than 30,000 €; this proportion was higher among neurology residents going up to 85% and lower among neurologists at around 67% (Table 2). A gender gap could be identified with 44% of women belonging to a low-income group (<15,000 €) as compared with 26.5% of men (eTable 10), in almost all career stages, except for PhD in Neurology. We took advantage of a multivariate analysis to identify factors associated with the low-income group, thus showing that female sex and geographic region (Eastern or other) are predicting factors for this outcome (eTable 11).

**Figure 2:**
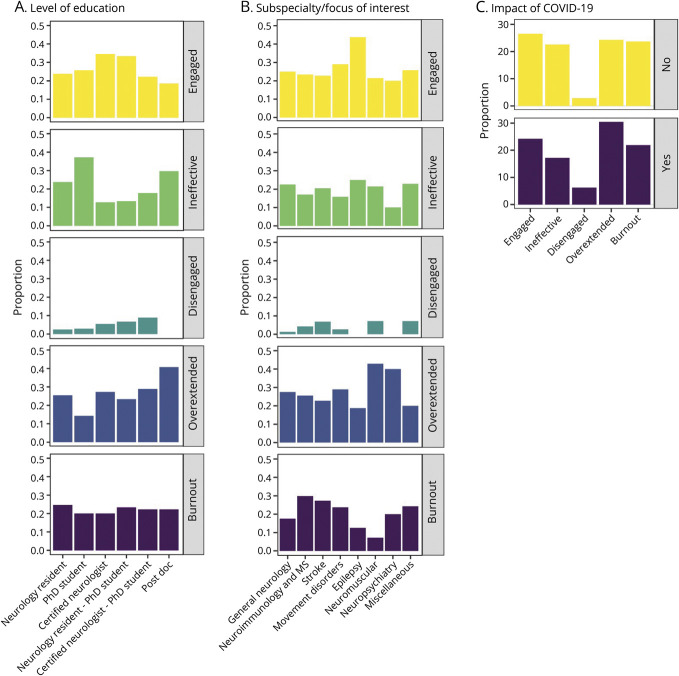
Burnout Profiles Based on Level of Education, Subspecialty, and COVID-19 Pandemic (A, B) Distribution of burnout profiles according to level of education achieved (A) and subspecialty of interest (B). (C) Impact of COVID-19 on burnout profile distribution expressed as proportion of profiles. 3D-WFI = Three‐Dimensional Work Fatigue Inventory; COVID-19 = coronavirus disease 2019; PHQ-9 = Patient Health Questionnaire-9.

The most popular topics, subspecialties of interest, or research focuses were general neurology (25.1%), neuroimmunology (14.7%), vascular neurology (13.8%), and movement disorders (11.9%) (Figure 2B).

### Distribution of Burnout Profiles Among EAN-RRFS Members

The MBI analysis revealed that 73.9% of responders showed perturbations in at least 1 burnout dimension (emotional exhaustion, cynicism, efficacy). Indeed, 22.6% of the responders fulfilled the criteria for a full-blown burnout, while the remaining responders were overextended (26.1%), ineffective (21.0%), disengaged (4.1%), or engaged (26.1%) (Figure 1, A and B).

Countries with the highest proportion of engaged responders belonged predominantly to Western Europe (39.7%, *p* = 0.01); overextended profiles were predominantly found in individuals working in Southern Europe (35.4%, *p* = 0.03) (eTables 1, 2, and eFigure 1B).

The proportion of burnout, disengaged, overextended, and engaged responders did not correlate with the country response rate (eFigure 1, C–G). We found a negative correlation between the proportion of ineffective responders and country response rate (*R*^2^ = 0.34, *p* < 0.01); however, this association remained of unknown significance considering the paucity of data on this profile.^9^

Burnout profiles were similarly distributed among different career paths (eTable 3 and Figure 2A) and subspecialty of interest (eTable 4, Figure 2B). The COVID-19 pandemic did not modify the distribution of burnout profiles in affected participants (eTable 5, Figure 2C).

### Factors Associated With Burnout

**Figure 3:**
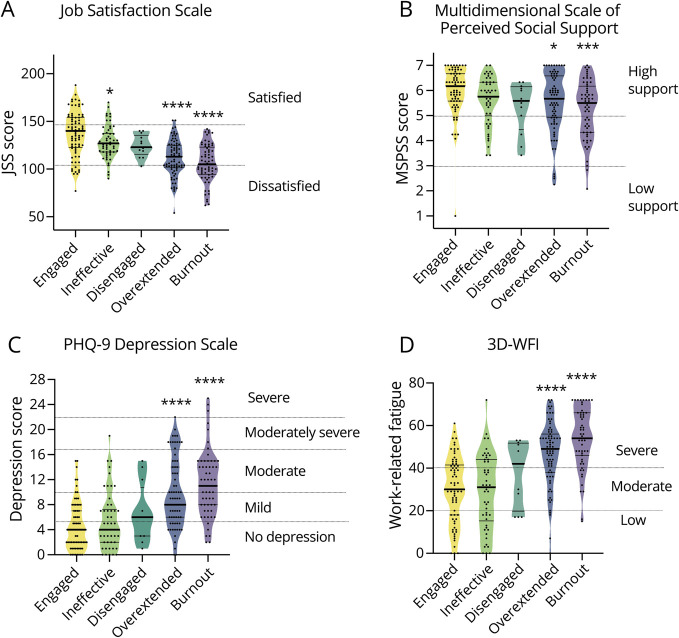
Multidimensional Profiling of Factors Associated With Burnout Profiles (A) Job Satisfaction Survey (JSS) score of distinct burnout profiles. Score lower than 108 express dissatisfaction, while scores higher than 144 identify satisfied responses. (B) Multidimensional Scale of Perceived Social Support (MSPSS) score of distinct burnout profiles. A mean scale score lower than 3 is considered as low support while a score of higher than 5 is considered as high support. (C) Patient Health Questionnaire 9 (PHQ-9) as depression scale for distinct burnout profiles. A score <4 indicates absence of depression, a score of 5–9 indicates mild depression, a score of 10–14 indicates moderate depression, a score of 15–19 indicates moderately severe depression, and a score 20 indicates severe depression. (D) Three-Dimensional Work Fatigue Inventory (3D-WFI) for distinct burnout profiles. A total score 40 indicates severe fatigue, a score between 20 and 40 indicates moderate fatigue, while a score <20 indicates low fatigue. Horizontal lines within the violin plots (A–D) indicate median (thick) and quartiles (thin), ****p < 0.0001, ***p < 0.001, **p < 0.01, *p < 0.05; ns, not significant by one-way analysis of variance with the Dunnett test (A–F) and the Tukey test (G–I) for multiple comparisons.

We detected a lower score in Job Satisfaction in responders with burnout, overextended, and ineffective profile as compared with engaged profile (Figure 3A and Table 3).

**Table 3:** Factors Associated With Burnout Spectrum

	Engaged	Ineffective	Disengaged	Overextended	Burnout	*p* Value
JSS score (n = 292)						
Mean	137.2	128.1	124.5	111.5	106.0	<0.001
Median	140.0	127.0	123.0	113.0	105.0	
SD	23.5	17.1	11.5	18.7	20.0	
MSPSS score (n = 269)						
Mean	6.0	5.6	5.3	5.5	5.2	0.002
Median	6.2	5.8	5.6	5.7	5.5	
SD	1.0	0.9	1.0	1.2	1.2	
PHQ-9 score (n = 269)						
Mean	4.6	5.5	6.8	9.8	11.3	<0.001
Median	4.0	4.0	6.0	8.0	11.0	
SD	3.8	4.5	4.6	5.4	5.2	
3D-WFI score (n = 269)						
Mean	30.1	29.2	37.5	46.9	53.9	<0.001
Median	30.0	31.0	42.0	49.0	54.0	
SD	15.2	17.3	14.9	15.1	14.0	
WSAS score (n = 269)						
Mean	10.2	12.1	15.0	19.3	22.5	<0.001
Median	8.0	12.0	16.0	19.0	23.0	
SD	7.7	8.4	10.2	9.3	8.7	
LCI score (n = 258)						
Mean	235.4	276.4	195.6	257.6	256.5	0.611
Median	188.5	199.0	205.0	233.0	207.5	
SD	167.5	238.2	109.7	170.3	167.9	
Hours per week (n = 314)						
Mean	48.0	44.3	44.8	51.7	54.2	<0.001
Median	48.0	45.0	48.0	50.0	55.0	
SD	14.4	13.3	13.6	11.8	12.6	
Nights on call per month (n = 314)						
Mean	2.6	2.3	3.7	2.3	3.5	0.013
Median	2.5	2.0	4.0	2.0	3.5	
SD	2.3	2.3	2.0	2.5	2.8	
Weekend on call per month (n = 314)						
Mean	1.4	1.6	1.6	1.6	1.8	0.524
Median	1.0	2.0	2.0	2.0	2.0	
SD	1.2	1.3	1.0	1.2	1.3	

Abbreviations: 3D-WFI = Three‐Dimensional Work Fatigue Inventory; JSS = Job Satisfaction Survey; LCI = Life Change Index Scale; MSPSS = Multidimensional Scale of Perceived Social Support; NA = not applicable; PHQ-9 = Patient Health Questionnaire-9; WSAS = Work and Social Adjustment Scale.

Among the subitems evaluated through the Job Satisfaction Survey (JSS), a lower score for communication (eFigure 2D), salary (eFigure 2E), supervision (eFigure 2G), fringe benefits (eFigure 2H), contingent rewards (eFigure 2I), and operating conditions (eFigure 2L) was found in both the burnout and overextended profile, while a lower satisfaction about promotion chances was reported by overextended responders (eFigure 2F). Burnout, overextended, and ineffective responders were also dissatisfied about the nature of their work and their coworkers (eFigure 2, J and K).

In addition, burnout and overextended individuals reported a reduced social support in contrast to members with an engaged profile (Figure 3B). They also displayed higher level depression (Figure 3C), work-related fatigue (Figure 3D), and impairment in work and social adjustments (eFigure 2A).

Responders within the burnout spectrum did not show a higher rate of stressful events (eFigure 2B).

Working hours were significantly higher among responders in burnout as compared with engaged ones (eFigure 2C). In addition, responders in burnout reported a larger number of night shifts per month as compared with colleagues fitting to ineffective and overextended profiles, while showing a similar number of weekend shifts per month (Table 2).

### Comparison of Burnout Rates and MBI Dimensions Between the United States and Europe

In previous studies on US neurology residents,^12^ fellows, and neurologists,^16^ burnout was defined by the presence of high scores on the emotional exhaustion or cynicism. To compare our data with these investigations, we aggregated the corresponding burnout profiles (burnout, overextended, and disengaged) and we also adapted the critical boundaries for the cynicism dimension because a lower threshold (≥10) was adopted in US studies, thus matching their burnout definition which is here defined as “burnout experience.”

We found that the prevalence of burnout experience is higher among US neurology residents (73.5%) than their European peers (52.5%). Moreover, the prevalence of burnout experience in European junior neurologists (58.2%) was similar to those of US neurology fellows (55.0%) and US neurologists (60.5%) (eFigure 3A).

Indeed, concerning the emotional exhaustion and cynicism dimensions, European neurology residents and junior neurologists showed lower median scores than US neurology residents, neurology fellows, and neurologists (eFigure 3, B and C). However, regarding the efficacy dimension, European participants obtained lower scores than their US colleagues (eFigure 4D), therefore suggesting a lower prevalence of the ineffective profile in the United States, although this aspect was not formally explored in their studies.

### Impact of Career Stage and Geographic Region

There was a higher JSS score among PhD students as compared with neurology residents, while career position did not affect other variables such as MSPSS, PHQ-9, 3D-WFI, WSAS, and LCI (eTable 6).

Western European responders showed higher JSS scores as compared with Eastern European and Southern participants (eTable 7).

The work-life balance impairment measured via the WSAS score was greater in Western Asiatic countries with partial territory in Southern Europe (Turkey) and Eastern Europe (Georgia), collectively defined as “other” in this study (eTable 7).

The weekly working hours reported by Southern European and Western Asiatic responders were higher than those reported by Eastern European countries (eTable 7). There was a lower number of night shifts per month in Western, Eastern, and Southern European responders as compared with Western Asiatic countries (eTable 7).

Reagrding workload, European neurology residents reported 50 hours per week (median) while US neurology residents^12^ reported 70 hours per week (median) (eFigure 3E). Similarly, junior European neurologists reported fewer working hours (40 hours per week, median) than their US colleagues (60 hours per week for fellows and 55 hours per week for neurologists, median, eFigure 3E).^12,16^

In addition, European neurology residents and junior neurologists reported fewer nights on call than their US peers (eFigure 3F).^12,16^

Responders from different geographic regions showed similar scores regarding MSPSS, PHQ-9, 3D-WFI, and LCI and similar numbers of weekend shifts per month (eTable 7).

### Impact of Academic Environment on Burnout Profiles

The burnout rate was 21% in colleagues working in a university hospital or laboratory, as compared with 29% working in a nonacademic environment (eTable 8).

Conversely, the engaged profile represented 27% of all colleagues working in an academic environment while 21% for nonacademic workplace (eTable 8).

Colleagues working in nonacademic environment showed higher scores in the emotional exhaustion and cynicism scale (eTable 9).

### Factors Predicting Burnout or Engaged Profiles in Multivariate Analysis

The multiple logistic regression analysis identified factors associated with burnout profile, thus considering as risk factors the number of night shifts per month, higher work-related fatigue, and as relieving factors higher scores in the JSS (Table 4, eFigure 5).

**Table 4:** Burnout Profile Multivariate Analysis

Model	Variable	Estimate	OR	95% CI	*p* Value	*p* Value summary
Burnout profile	Night	0.218	1.244	1.084–1.436	0.002	**
	JSS score	−0.023	0.978	0.959–0.996	0.017	*
	3D-WFI score	0.052	1.054	1.024–1.088	<0.001	***
Engaged profile	Certified neurologist	1.022	2.779	1.210–6.444	0.016	*
	JSS score	0.036	1.037	1.020–1.056	<0.001	***
	MSPSS	0.439	1.552	1.089–2.290	0.020	*
	PHQ-9	−0.097	0.907	0.823–0.994	0.043	*

Abbreviations: 3D-WFI = Three‐Dimensional Work Fatigue Inventory; JSS = Job Satisfaction Survey; MSPSS = Multidimensional Scale of Perceived Social Support; OR = odds ratio; PHQ-9 = Patient Health Questionnaire-9.

Variables in the multivariate logistic model for burnout profile as outcome: burnout profile, JSS, MSPSS, night shift per month, 3D-WFI, PHQ-9, being a certified neurologist; variables in the multivariate logistic model for engaged profile as outcome: engaged profile, JSS, MSPSS, night shift per month, 3D-WFI, PHQ-9, being a certified neurologist.

Asterisks denote statistical significance significance: *p < 0.05; **p < 0.01; ***p < 0.001.

Multivariate analysis revealed that the career stage certified neurologist, low score in PHQ-9, high scores in the JSS, and in the MSPSS are considered as factors predicting the engaged profile (Table 4, eFigure 5).

## Discussion

Over the past few decades, the medical community is facing an alarming increase in burnout rate and deterioration in professional well-being. Restraining the impact of burnout represents a major challenge for medical education and public health.^24^ This issue is further exacerbated by the shortage in supply of neurologists and increase in their demand in the United States and Europe for the upcoming years as well as imbalance in subspecialty choice.^1,14,25^

Our profile-based approach^9^ revealed that 73.9% of the responders experienced a dysfunctional phenotype within the burnout spectrum: burnout (22.6%), overextended (26.1%), ineffective (21.0%), and disengaged (4.1%). Dissecting the distinct burnout phenotypes provided a unique opportunity for delineating appropriate mitigating strategies and avoiding an overestimation of this phenomenon among European neurology residents, junior neurologists, and research fellows.

Previous studies on US neurology residents, fellows, and certified neurologists have adopted a dichotomic definition of burnout, thus considering a perturbation in a single MBI dimension as burnout.^12,16,26^ This approach might overestimate the prevalence of burnout; however, even adopting the same definition, the prevalence of burnout symptoms was lower in European neurology residents than in US peers.

Burnout has a multifactorial etiology emerging from a combination of causative factors which are tailored to the specific workforce population. Understanding this dangerous combination of poisoning factors requires a deep understanding of the dysfunctional work-life integration.

Job dissatisfaction is a leading force of burnout, encompassing different aspects such as salary, supervision, coworkers, and the nature of work among other features. Moreover, we highlighted a gender gap with 73.5% of men earning more than 15,000 € as compared with 56% of women in almost all career stages of neurology. The earning disparity between male and female neurologists represents also one of the largest pay gaps for any medical specialty in the United States.^14^ Multivariate analysis also revealed that working in Eastern Europe or Western Asiatic countries might contribute to these earning disparities.

We showed that night shifts are another independent risk factor for burnout, thus corroborating the evidence emerging from previous^12,16,27^ and highlighting the importance of appropriate working schedules^28^ limiting work-related fatigue.^21^ Weekly working hours were significantly higher among responders in burnout as compared with engaged participants. However, European neurology residents and junior neurologists reported fewer working hours than their US colleagues.^12^

In addition, we showed that both burnout and overextended profiles are at high risk for depression as compared with engaged profiles. Burnout and depression are 2 distinct nosographic entities, but they can both exist in presence of a dysfunctional work environment.^27,29^ This combination might be particularly worrisome given the increased risk of suicide^28^ which represents a leading cause of mortality among young physicians especially during the first years of residency.^30^

Burnout profiles were distributed in a peculiar pattern according to the geographic region with engaged participants working predominantly in Western Europe, while overextended participants were working in Southern Europe, thus indicating some protective or distressful factors intrinsic to the specific neurology residency programs and workplace regulations. Indeed, country-specific factors might modulate burnout prevalence, considering the diversity of higher education systems. Despite sharing a core of knowledge and skills, European neurology residency programs have a certain degree of heterogeneity regarding duration and mandatory rotations,^25^ while doctoral programs are defined by numerous regulations in Europe,^31^ therefore exposing residents and PhD students to different working environments. Moreover, a wide range of different health care systems run at individual national levels, therefore also modifying the burden of clerical tasks for neurology residents and junior neurologists. Nevertheless, future studies are needed to identify country-specific risk factors for burnout.

The COVID-19 pandemic has transformed health care delivery.^32^ Despite nearly 44% of the participants reported an increased workload related to this pandemic,^33,34^ we did not detect an increase in burnout profiles among the affected participants in our study. Indeed, a previous study has shown that the specialties mostly affected by this pandemic (emergency medicine, critical care, hospital medicine, and infectious diseases) did not show an increase in symptoms of burnout.^35^ In addition, the pandemic might also have contributed to a constructive adaptation by connecting some physicians to meaning and purpose in their work,^36^ thus likely alleviating the occupational distress.

Our study revealed that the engaged profile was promoted by higher levels of professional satisfaction, social support, and a more advanced career stage such as certified neurologists.

Although the lower burnout prevalence in junior neurologists might appear surprising because of their recent certification, the different work environment represents a possible explanation given that residents and junior neurologists differ regarding workload (working hours and weekend shifts) and need of supervision. Indeed, insufficient supervision has been associated with burnout and poor psychological well-being,^37^ therefore ameliorating the clinical learning environment is fundamental to improve mental health. In addition, the protective effect of seniority was also identified among practicing neurologists^16^ and fellows as compared with residents.^12^

Moreover, emotional exhaustion among neurologists increases at the beginning of their career and then decreases as neurologists get more advanced. Similarly, the cynicism dimension of the MBI decreases with advancing age.^38^

The academic environment represented a protective factor for burnout, thus corroborating similar findings from other authors.^12^

Previous studies have shown a protective effect of social support on burnout rates among health care professionals.^39^ In this study, we show that individuals fitting to burnout and overextended profiles lack appropriate resources regarding supportive social interactions from family and friends according to the MSPSS scale. These individuals also reported signs of functional impairment not only in the professional sphere but also in their private life affecting home management, leisure activities, and social relationships as revealed by the WSAS scale, therefore suffering from insufficient work-life integration.^35^

The social and economic impact of burnout is substantial, considering that the costs related to physician turnover and reduced clinical hours ranged from $2.6 to $6.3 billions in the United States.^38^ This led to proposals of various strategies aiming at preventing this phenomenon, treating affected individuals,^40^ or promoting physician wellness.^41^

Given the multifaceted nature of burnout experience, preventing strategies should be tailored to the distinct dysfunctional burnout profile.

Overextended, disengaged, and ineffective represent transitional states between the engaged and burnout continuum and could be considered as either steps toward a full-blown burnout or its aftermath^9^ (eFigure 4).

Overextended physicians are devoted to their job from which they get a sense of professional efficacy and satisfaction; however, they can become exhausted by long working hours, night shifts without sufficient recovery, excessive clerical tasks, and disrupted sleep-wake cycles. Therefore, workload-oriented interventions could be a mitigating measure for overextended physicians.^42^

The disengaged profile is mainly characterized by the presence of cynicism which is linked to the poor quality of social relationships at work and the lack of critical resources, thus leading to a reduced job satisfaction and performance.^43^ Thus, these individuals would benefit from interventions designed to improve social relationships among coworkers and workplace civility among health care providers.^10^

Ineffective individuals experience a loss of confidence in their capabilities, feelings of inadequacy, and failure. This sense of inefficacy could be responsive to an improved autonomy-supportive learning environment with a culture of supportive collegiality.^44^ In addition, the adoption of mentorship programs involving senior colleagues might be helpful in providing guidance and infuse meaning in the clinical and academic learning environment as well as promoting the value of academic teaching.^45^

All these strategies should be implemented simultaneously to prevent the development of a full-blown burnout. Our multivariate analyses indicate that relieving work-related fatigue, promoting professional satisfaction, reducing the frequency of night shifts, and encouraging a stronger social support might be an effective measure to promote the transition toward the engaged profile.

Further research combining both evaluation of burnout profiles and dedicated interventions would be particularly useful in understanding and restraining this phenomenon.

Future studies are also needed to track the trajectories of physicians currently affected by burnout to evaluate a transition among distinct burnout profiles and delineate their driving factors.

Our study is subject to several limitations. First, this study has a relatively small sample size of 332 participants of 1,439 RRFS members. Despite being similar to other burnout surveys,^2,46-48^ the response rate of 23.1% in this study is lower than that achieved in previous studies in neurology residents and neurologists in the United States.^12,16^ It is likely that the extensive characterization incorporating numerous validated psychometric scales might have contributed to the lower response rate.

Because 80% of the responders were working in academic environments, the RRFS community might not be a fair representation of neurology residents all over Europe given their high involvement in academia, and this might underestimate burnout prevalence.

Given the cross-sectional design of the survey, we were not able to determine causality for the observed associations.

For the first time, our study uses a multifaceted approach allowing the discrimination of 5 distinct burnout profiles and providing a more reliable estimate of the full-blown burnout experience and other transitional states. Although this profile-based approach allows a deep understanding of the multifaceted burnout experience, it is possible that each profile contains an heterogenous group of individuals.

The present data document a high prevalence of burnout experience among European neurology residents, junior neurologists, and research fellows.

The profile-based approach adopted in this survey captured its multifaceted nature, therefore providing an appropriate estimate for full-blown burnout (22.6%) and other transitional states emerging either as its precursors or sequelae (51.2%) which can benefit from a tailored mitigating strategy.

Our study indicates that the engaged-burnout continuum can be shifted toward burnout because of numerous night shifts, work-related fatigue, and job dissatisfaction, while social support, seniority, and lack of depression are factors promoting the engaged profile.

Overextended, disengaged, and ineffective profiles represent transitional states requiring tailored mitigating strategies. Indeed, overextended individuals are most responsive to workload-oriented interventions while physicians fitting the disengaged profile may benefit from interventions designed to improve social relationships among coworkers. Because ineffective individuals manifest a loss of confidence in their capabilities, reinforcing mentorship and improving the learning environment might be beneficial.

A dichotomic definition of burnout relying on the perturbation of a single MBI dimension might have overestimated its prevalence among neurology residents and neurologists in previous studies conducted in the United States.^12,16^ However, even adopting the same definition, the prevalence of burnout symptoms was lower in European neurology residents than in US colleagues.

Because this issue represents an occupational hazard for both patients and physicians, coordinated interventions are required and can be obtained only by the collaborative efforts of local clinical and academic coordinators, national health care systems, and European organizations.

## Appendix Authors

**Table:**

Name	Location	Contribution
Giovanni Di Liberto, MD, PhD	Department of Clinical Neurosciences, Centre Hospitalier Universitaire Vaudois and University of Lausanne, Switzerland	Drafting/revision of the manuscript for content, including medical writing for content; major role in the acquisition of data; study concept or design; analysis or interpretation of data
Giuseppina Baldizzi, MSc	Department of Clinical Neurosciences, Centre Hospitalier Universitaire Vaudois and University of Lausanne, Switzerland	Drafting/revision of the manuscript for content, including medical writing for content; major role in the acquisition of data; study concept or design; analysis or interpretation of data; additional contributions: co-first author
Vanessa Carvalho, MD	Department of Neurosciences and Mental Health (Neurology), and Centro de Estudos Egas Moniz, Faculdade de Medicina, Universidade de Lisboa, Portugal	Drafting/revision of the manuscript for content, including medical writing for content; study concept or design
Luca Cuffaro, MD	Department of Health Sciences, Clinical Neurology Unit, ASST Santi Paolo & Carlo, Milan, Italy	Drafting/revision of the manuscript for content, including medical writing for content; study concept or design
Anna Sauerbier, MD	Department of Neurology, Faculty of Medicine and University Hospital Cologne, University of Cologne, Germany	Drafting/revision of the manuscript for content, including medical writing for content; study concept or design
Lisa Klingelhoefer, MD	Department of Neurology, Technical University Dresden, Germany	Drafting/revision of the manuscript for content, including medical writing for content; study concept or design
Nina Vashchenko, MD	University Hospital of Neurological Disorders, Sechenov First Moscow State Medical University, Russia	Drafting/revision of the manuscript for content, including medical writing for content
Lucia Pavlakova	European Academy of Neurology, Head Office, Vienna, Austria	Drafting/revision of the manuscript for content, including medical writing for content; major role in the acquisition of data
Anja Sander	European Academy of Neurology, Head Office, Vienna, Austria	Drafting/revision of the manuscript for content, including medical writing for content; major role in the acquisition of data
Marianne de Visser, MD, PhD	Department of Neurology, Academic Medical Center, Amsterdam, the Netherlands	Drafting/revision of the manuscript for content, including medical writing for content; analysis or interpretation of data
Paul A.J.M. Boon, MD, PhD	Department of Neurology, Ghent University Hospital, Belgium	Drafting/revision of the manuscript for content, including medical writing for content; analysis or interpretation of data
Elena Moro, MD, PhD	Division of Neurology, CHU of Grenoble, Grenoble Institute of Neurosciences, Grenoble Alpes University, France	Drafting/revision of the manuscript for content, including medical writing for content; analysis or interpretation of data
Claudio Bassetti, MD	Department of Neurology, Inselspital, University of Bern, Switzerland	Drafting/revision of the manuscript for content, including medical writing for content; study concept or design; analysis or interpretation of data

## Glossary

3D-WFI: Three-Dimensional Work Fatigue Inventory
COVID-19: coronavirus disease 2019
EAN: European Academy of Neurology
JSS: Job Satisfaction Survey
LCI: Life Change Index Scale
MBI: Maslach Burnout Inventory
MBI-HSS: MBI-Human Services Survey
MSPSS: Multidimensional Scale of Perceived Social Support
PHQ-9: Patient Health Questionnaire-9
RRFS: Resident and Research Fellow Section
WSAS: Work and Social Adjustment Scale
